# The promise of mRNA vaccines: a biotech and industrial perspective

**DOI:** 10.1038/s41541-020-0159-8

**Published:** 2020-02-04

**Authors:** Nicholas A. C. Jackson, Kent E. Kester, Danilo Casimiro, Sanjay Gurunathan, Frank DeRosa

**Affiliations:** 1 Coalition for Epidemic Preparedness Innovations (CEPI), Gibbs building, 215 Euston Road, Bloomsbury, London, NW1 2BE UK; 2 Sanofi Pasteur, 1 Discovery Dr, Swiftwater, PA 18370 USA; 3 Translate Bio, 29 Hartwell Ave, Lexington, MA 02421 USA

**Keywords:** Vaccines, Infectious diseases

## Abstract

mRNA technologies have the potential to transform areas of medicine, including the prophylaxis of infectious diseases. The advantages for vaccines range from the acceleration of immunogen discovery to rapid response and multiple disease target manufacturing. A greater understanding of quality attributes that dictate translation efficiency, as well as a comprehensive appreciation of the importance of mRNA delivery, are influencing a new era of investment in development activities. The application of translational sciences and growing early-phase clinical experience continue to inform candidate vaccine selection. Here we review the state of the art for the prevention of infectious diseases by using mRNA and pertinent topics to the biotechnology and pharmaceutical industries.

## Introduction

Continued growth in the vaccine business is expected based on expanded coverage, improved existing products, and new vaccines. Among other factors, manufacturing must change to support growth. Capital-rich investment in fixed facilities that commit to a given form of production for a given target poses significant costs and challenges to vaccine manufacturers if there is a major change in strategy. Long lead times in manufacturing, and potentially hundreds of complex process steps, all complicate capabilities. Vaccine companies, like the biotech industry, desire novel production methods out of a need for efficiency that reduce the cost of goods, shorten time to licensure, and respond quicker to disease outbreaks. mRNA-based vaccines hold the promise to revolutionize the field by addressing current manufacturing challenges and offering novel vaccine compositions.

Assuming that mRNA vaccines will be proven clinically efficacious and safe, one of the central advantages hinges on rapidity of manufacture. Within weeks, clinical batches can be generated after the availability of a sequence encoding the immunogen. The process is cell-free and scalable. Of paramount advantage, a facility dedicated to mRNA production should be able to rapidly manufacture vaccines against multiple targets, with minimal adaptation to processes and formulation. In addition, new targets requiring multi-antigen approaches will benefit from the speed in which mRNA can render multiple constructs.

Beyond manufacturing advantages, mRNA technology is impacting vaccine discovery and research. Expression may be possible for complex proteins that are difficult or impossible to generate with current expression systems.^1^ mRNA constructs can also be used to express potent monoclonal antibodies for novel immunoprophylaxis.^2^ Here we review the state of the art in mRNA constructs and delivery technologies for the prevention of infectious diseases, and a review of pertinent topics to the biotechnology and pharmaceutical industries.

## “State-of-the-art” mRNA constructs and delivery technologies

**Fig. 1:**
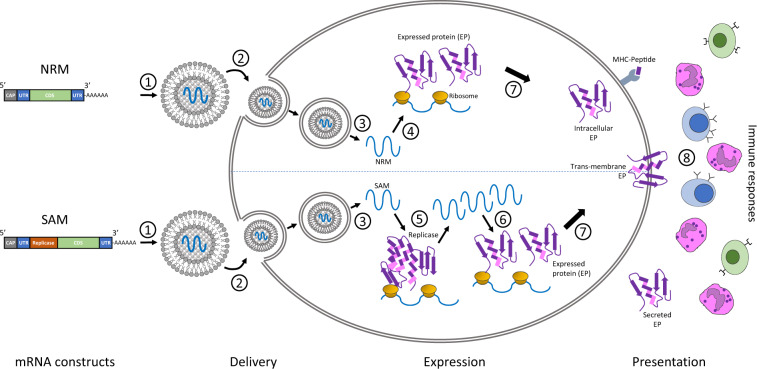
Two categories of mRNA constructs are being actively evaluated. Non-replicating mRNA (NRM) constructs encode the coding sequence (CDS), and are flanked by 5′ and 3′ untranslated regions (UTRs), a 5′-cap structure and a 3′-poly-(A) tail. The self-amplifying mRNA (SAM) construct encodes additional replicase components able to direct intracellular mRNA amplification. (1) NRM and SAM are formulated in this illustration in lipid nanoparticles (LNPs) that encapsulate the mRNA constructs to protect them from degradation and promote cellular uptake. (2) Cellular uptake of the mRNA with its delivery system typically exploits membrane-derived endocytic pathways. (3) Endosomal escape allows release of the mRNA into the cytosol. (4) Cytosol-located NRM constructs are immediately translated by ribosomes to produce the protein of interest, which undergoes subsequent post-translational modification. (5) SAM constructs can also be immediately translated by ribosomes to produce the replicase machinery necessary for self-amplification of the mRNA. (6) Self-amplified mRNA constructs are translated by ribosomes to produce the protein of interest, which undergoes subsequent post-translational modification. (7) The expressed proteins of interest are generated as secreted, *trans*-membrane, or intracellular protein. (8) The innate and adaptive immune responses detect the protein of interest.

The core principle behind mRNA as a technology for vaccination is to deliver the transcript of interest, encoding one or more immunogen(s), into the host cell cytoplasm where expression generates translated protein(s) to be within the membrane, secreted or intracellularly located. Two categories of mRNA constructs are being actively evaluated: non-replicating mRNA (NRM) and self-amplifying mRNA (SAM) constructs (Fig. 1). Both have in common a cap structure, 5′ and 3′ untranslated regions (UTRs), an open-reading frame (ORF), and a 3′ poly(A) tail.^3^ SAM differs with the inclusion of genetic replication machinery derived from positive-stranded mRNA viruses, most commonly from alphaviruses such as Sindbis and Semliki-Forest viruses.^4,5^ Generally, the ORF encoding viral structural proteins is replaced by the selected transcript of interest, and the viral RNA-dependent RNA polymerase is retained to direct cytoplasmic amplification of the replicon construct. The potential merits of NRM versus SAM will be addressed later.

The manufacturing process begins with the generation of a plasmid DNA (pDNA) containing a DNA-dependent RNA polymerase promoter, such as T7,^6^ and the corresponding sequence for the mRNA construct. The pDNA is linearized to serve as a template for the DNA-dependent RNA polymerase to transcribe the mRNA, and subsequently degraded by a DNase process step. The addition of the 5′ cap and the 3′ poly(A) tail can be achieved during the in vitro transcription step^7,8^ or enzymatically after transcription.^9^ Enzymatic addition of the cap can be accomplished by using guanylyl transferase and 2′-O-methyltransferase to yield a Cap 0 (^N7Me^GpppN) or Cap 1 (^N7Me^GpppN^2′-OMe^) structure, respectively, while the poly-A tail can be achieved through enzymatic addition via poly-A polymerase.

Purification is a crucial next step, which can be achieved with the application of high-pressure liquid chromatography (HPLC).^10^ The resultant drug substance is then formulated into drug product and released based on sterility, identity, purity, and potency testing. These processes allow Good Manufacturing Practise (GMPs) facilities to switch to a new vaccine within a very short period of time, given that the reaction materials and vessels are the same.

**Fig. 2:**
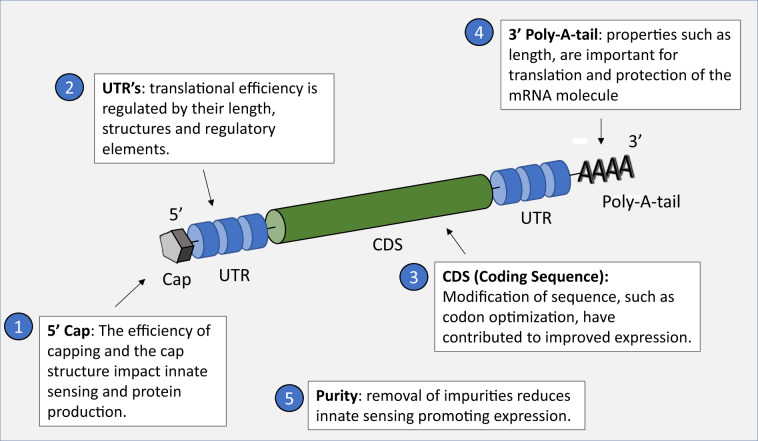
Critical quality attributes (CQAs) have been identified that dictate the performance of the mRNA construct to express the gene of interest efficiently. Five principal CQAs include 5′ capping efficiency and structure; UTR structure, length, and regulatory elements; modification of coding sequence; poly-A-tail properties; mRNA purity.

The design of a mRNA construct for vaccination, once released into the cytoplasm of a cell, is to efficiently utilize the translational machinery of the host cell to generate a sufficient quantity of the encoded immunogen that is presented appropriately to the immune system. Across the field, several critical quality attributes have been, and continue to be, the focus of efforts to maximize gene expression (Fig. 2). First, the purity of the mRNA is a crucial determinant of yields, and it is known that the DNA-dependent RNA polymerases yield smaller oligoribonucleotide impurities as a result of abortive initiation events,^11^ as well as double-stranded (ds) RNA generated by self-complementary 3′ extension,^12^ which can result in type I interferon and inflammatory cytokine production through pattern recognition receptors. Karikó et al.^13^ demonstrated that removal of contaminants in mRNA preparations reduced innate immune responses and resulted in significantly higher levels of reporter protein expression in vitro.

Second, the 5′ and 3′ UTR regions are important for maximizing gene expression. The length of the 3′ UTR,^14^ 5′ UTR structures, and regulatory elements in both UTRs^15^ all impact efficiency. Third, the 5′ 7-methylguanosine (m7G) cap of the mRNA molecule, linked via a triphosphate bridge to the first transcribed nucleotide, is essential for efficient translation, and blocks 5′–3′ exonuclease-mediated degradation. The specific cap structure plays a critical role in both protein production and immunogenicity, with incomplete capping (5′ triphosphate) and Cap 0 structures shown to stimulate RIG-1.^16–18^ In addition, 2-O′-unmethylated capped RNA can be sequestered by cellular IFN-induced proteins with tetratricopeptide repeats (IFIT1) that prevent the initiation of translation,^19^ or detected by the cytoplasmic RNA sensor MDA5.^20^ Manufacturers of mRNA vaccines pay careful attention to the choice of enzyme and reaction conditions, in order to catalyze the highest percentage of cap formation. Fourth, the poly (A) tail and its properties such as length, are crucial for translation and protection of the mRNA molecule.^21,22^

Last, codon optimization and modification of nucleotides have contributed to translation efficiency. For example, optimization of guanine and cytosine (GC) content can have a significant impact,^23^ and has been well established with DNA vaccines. The innate immune activation to mRNA can also influence its utility as a delivery system. The use of modified nucleosides, such as pseudouridine or N-1-methylpseudouridine to remove intracellular signaling triggers for protein kinase R (PKR) activation, resulted in enhanced antigen expression and adaptive immune responses.^24–26^ It has been demonstrated that successful protein production, minimal undesired inflammatory responses, and systemic adaptive immune responses could be achieved preclinically by using unmodified mRNA^27^ through a combination of optimizing the coding sequence and removal of any unwanted inflammatory impurities.^28,29^ Ultimately, comparative immunogenicity between these approaches require studies that control all potential factors, including the delivery system. Human studies “head-to-head” comparing modified and unmodified nucleoside mRNA constructs would confirm clinically relevant differences, if any. Similarly, controlled comparisons of the aforementioned NRM and SAM are needed to determine any distinctions. Until then, all the approaches—modified, unmodified, NRM, SAM, and combinations thereof—appear feasible, and are supported by preclinical data, although unmodified nucleoside constructs may be desirable for manufacturing efficiency and transcriptional fidelity.^30,31^

In addition to optimizing an mRNA construct, and of quintessential importance, is the delivery of the mRNA vaccine from the bolus at the injection site into the cytoplasm of cells for the initiation of translation. As mRNA is a transient molecule by nature that is susceptible to degradation primarily through nuclease activity, efficient protection is required.^32,33^ This has been an intense area of research in the field, for which lipid nanoparticle (LNP) formulations are currently emerging as a leading category.

LNP delivery systems serve multiple purposes in their applications. In addition to the aforementioned sustained stability imparted through protection from nuclease degradation, they also facilitate organ specificity, efficient cellular uptake, and provide endosomal escape properties that can enhance the successful delivery of the mRNA cargo to the cytoplasmic site of action.^34–36^ There have been numerous examples of successful delivery of mRNA by using LNPs for therapeutic^37–40^ as well as vaccine applications.^41–44^

Much of the focus of the continued development of such LNP carrier systems involves optimization of the ionizable lipid component, with particular focus on the acid dissociation constant (pKa) and fusogenic properties (both of the ionizable component as well as helper lipid[s]), which have been demonstrated to play key roles in efficient cytoplasmic entry and release of cargo.^45–48^ Next-generation LNPs may include specific targeting motifs for homing and uptake by professional antigen-presenting cells, such as dendritic cells (DC). Ligands for DC receptors could be embedded on the surface of the LNPs to target these cells and promote antigen presentation to the immune system.

## Biotech and industrial perspectives

### Perspective #1: Improved understanding of the molecular mechanisms of action will guide further improvements in mRNA constructs and formulations

Continual optimization and improvements toward developing the next generation of mRNA-based drugs are undoubtedly occurring. Sequence optimization within UTRs and coding regions of mRNA providing greater stability and/or potency can result in higher production of the desired antigen, potentially leading to a more favorable therapeutic index.^49,50^

Additional sites within the mRNA construct are available for optimization as well. Novel cap structures focused on base or sugar modifications have resulted in greater translational properties through increased ribosomal interaction or enhanced stability.^51^ Improved stability of mRNA can also be achieved through various modifications of the triphosphate bridge within the cap structure.^52–54^

Optimization of the carrier system can provide significant benefit as well. Substantial attention has been placed on the development of novel ionizable lipids and formulations, with improvements in cellular uptake, endosomal release, potency, and biodegradability.^55–57^ The combinations of all of these areas for optimization are near limitless, and success within any of these parameters can allow for beneficial effects and can provide novel approaches for vaccines to successfully prevent disease.

### Perspective #2: Translational sciences will inform preclinical and clinical studies to promote rapid downselection of constructs and formulations

A key aspect of vaccine development efforts is the goal of making early informed decisions, based on objective data that favor or disfavor a particular candidate. It is underappreciated in the field that multi-antigen vaccine approaches are a significant challenge in the decision-making process. For example, the LNP:mRNA mass ratio can be around 10:1 – 30:1. Thus, multi-antigen candidates necessitate a significant amount of LNP for a given dose. LNPs are known to have inherent adjuvant properties.^58^ Therefore, safety and tolerability may limit multi-antigen approaches, and here translational sciences are crucial for development.

There are a variety of new translational medicine tools that can be leveraged in evaluating immunity, which include in vitro human immune system models and the related organoids to improve the predictability of clinical results.^59^ Systems biology techniques can help in the understanding of fine differences between various NRM and SAM vaccine sequences, as well as serving as useful tools to frame sequence optimizations during iterative development schemes.^60^ Further, the use of functional assessments, like human challenge models,^61^ where the immunologic profiles of the participants and the specific details of the challenge strain(s) are well characterized—an approach mostly used to date in the evaluation of more traditional vaccines—provides an important and powerful method to obtain early decision-making data regarding the performance of an mRNA vaccine candidate. These tools are likely quintessential for development because current data have demonstrated a poor translation between preclinical and clinical studies with an mRNA pandemic influenza vaccine. In ferrets (ID, 2 × 50/100 mcg), nonhuman primates (ID/IM, 2 × 400 mcg), and humans (IM, 2 × 100 mcg), an H10N8-derived HA mRNA vaccine formulated with LNP elicited HAI titers in the range of 2000–8000, 10,000, and 70, respectively.^62,63^ Whether this lack of translation is a function of mRNA not having optimized quality attributes, suboptimal delivery or an inherent limitation of preclinical or in vitro translational models is not known. Understanding this is going to be key for further development.

### Perspective #3: Challenges ahead for clinical trials

While specific regulatory guidelines are lacking for the clinical development of mRNA-based vaccines, the following general principles, as outlined in overarching guidance documents, are generally sufficient to help facilitate the entry of candidate vaccines into early-phase clinical trials. At the time of writing, 12 clinical trials for mRNA-based infectious disease vaccines have been completed, or are at various stages of progression, building experience (see Table 1). All studies assess viral targets, many have been reviewed elsewhere recently.^64^

**Table 1:** Summary of clinical studies assessing infectious disease mRNA vaccines.

Disease target	Study stage	mRNA	Delivery formulation	Trial citation	Status	Organization	Ref.
RSV	Ph1	mRNA-1172	Merck proprietary formulation	Not known	Ongoing	Merck/Moderna	^70^
Rabies	Ph1	RG-SAM [CNE]	Cationic lipid formulation	NCT04062669	Ongoing	GSK	https://clinicaltrials.gov/ct2/show/NCT04062669
Chikungunya virus	Ph1	mRNA-1388	Not disclosed	Not known	Ongoing (interim results)	Moderna	^71^
Rabies	Ph1	CV7202	LNP	NCT03713086	Ongoing (interim results)	Curevac	^72^
Human metapneumovirus (hMPV) and parainfluenza type 3 (PIV3)	Ph1	mRNA-1653	LNP	NCT03392389	Ongoing (interim results)	Moderna	^73^
Influenza (H10N8)	Ph1	mRNA-1440	LNP	NCT03076385	Ongoing (interim results)	Moderna	^63^
Influenza (H7N9)	Ph1	mRNA-1851	LNP	NCT03345043	Ongoing (interim results)	Moderna	^63^
Cytomegalovirus (CMV); six valencies expressing a pentamer and gB	Ph1	mRNA-1647 mRNA-1443	LNP	NCT03382405	Ongoing (interim results for mRNA-1647)	Moderna	^74^
Zika virus	Ph1	mRNA-1893	LNP	NCT04064905	Ongoing	Moderna	^75^
Zika virus	Ph1	mRNA-1325	Not disclosed	NCT03014089	Completed	Moderna	^76^
Rabies virus	Ph1	CV7201	Protamine	NCT02241135	Completed	Curevac	^69^
RSV	Ph1	mRNA-1777	Not disclosed	Not known	Completed (not yet reported)	Merck/Moderna	^68^

The current focus from a clinical perspective is to optimize the benefit (immunogenicity and efficacy) while reducing the risk (safety) profile of a candidate mRNA vaccine by optimizing the quality attributes that dictate expression and/or augmenting delivery. It is clear that immune activation can be both advantageous and potentially detrimental, and has to be titrated accordingly. Thus, early-phase clinical trials need to be designed in a way to appropriately capture the inflammatory component intrinsic to all mRNA vaccines, given that several intracellular innate immune response sensors are activated by RNA.^65^ Elements include measuring administration site reactions such as pain, tenderness-associated systemic reactions such as fever and malaise, and routine biochemical laboratory parameters (e.g., serum electrolytes, liver function test, and CBC). These parameters, when followed closely, can be designed to develop enrollment pause rules in the event that severe tolerability issues are observed in a clinical trial.^66^ Detailed characterization of the immune response fully leveraging modern techniques such as transcriptomics and systems biology, in addition to traditional methods of immune monitoring, needs to be implemented. Several agency guidelines developed for the study of novel adjuvants in human subjects provide sufficient guidance that can be applied to mRNA candidate vaccines on how to monitor safety in early-phase clinical studies.^67^

The data from early-phase clinical studies, particularly around local and self-limiting systemic reactogenicity, have been mixed.^68,69^ In fact, reporting of human trials has generally concluded that new formulations are required to optimize the profile. However, these early claims need further confirmation, and in many cases, complete datasets are still awaited. As mentioned above, multi-antigen approaches will only complicate the issue of establishing acceptable tolerability.

Humoral elicited responses have been generally underwhelming, compared with the established potency in the field of protein or live attenuated vaccines.^63,69^ This indicates that much formulation work is still needed to achieve sufficient immunological potency of different vaccine candidates, while maintaining acceptable tolerability—but we can be encouraged by incremental progress to date. Furthermore, very limited data exist on repeat administration of mRNA vaccines in humans. These data are important as most vaccines generally require a booster dose.

As the field accrues more data from early-phase human studies, the focus of mRNA vaccines will shift from documenting local and systemic tolerability to capturing potential long-term safety. Unfortunately detecting safety signals for uncommon adverse events requires thousands of subjects. As with novel adjuvants, an adequate safety database to assure safety for candidate mRNA vaccines is likely to be in the tens of thousands range. Given that different manufacturers are pursuing different strategies to optimize their candidate vaccines, conclusions from one candidate may not be generalizable. Therefore, it is likely that each candidate vaccine will have to independently prove its risk/benefit profile that is favorable.

## Summary

The potential advantages of mRNA as a vaccine range from the discovery of immunogens to rapid response manufacturing. Currently, the field is pursuing two approaches: non-replicating and self-replicating constructs. A number of quality attributes, that dictate stability and efficiency of expression, continue to be an intense area of development. It is widely recognized that the delivery of the mRNA into the cytoplasm is equally important to successfully elicit a robust and durable immunity. As a result, much progress has been achieved with considerable focus on novel ionizable lipid formulations and the next generation of delivery systems.

The nature of mRNA technology allows rapid refinement with almost limitless combinations of derivatives in the pursuit of optimization. This necessitates the application of translational sciences to accelerate selection of the optimal construct and formulation for subsequent development. Clinical experience in the last 2 years is building upon the plethora of preclinical data generated. These trials have informed our understanding of the need to find the optimal balance between immune and inflammatory activation to establish an acceptable risk/benefit profile for a given vaccine. Whether clinical results from different sponsors will be generalizable for mRNA technologies and formulations remains to be determined.

Overall, with significant advances in mRNA biology, delivery, and manufacturing, the biotechnology and vaccine industries are poised for further investment in the development of novel products.
